# Community-based primary healthcare training for physiotherapy: Students’ perceptions of a learning platform

**DOI:** 10.4102/sajp.v75i1.471

**Published:** 2019-05-29

**Authors:** Vijaya Misra, Nomzamo Chemane, Stacy Maddocks, Verusia Chetty

**Affiliations:** 1 School of Health Sciences, University of KwaZulu-Natal, Durban, South Africa

**Keywords:** community-based primary healthcare, clinical training, physiotherapy, clinical education, clinical placements, South Africa

## Abstract

**Background:**

South Africa is faced with an overburdened public healthcare system and physiotherapists need to be equipped to address these challenges. Community-based primary healthcare clinical training (CBPHCT) offers physiotherapy students with learning opportunities to develop core competencies in order to address the needs of a disparate healthcare system.

**Objectives:**

To explore the experiences of physiotherapy students participating in a CBPHCT platform.

**Method:**

An explorative qualitative approach was adopted, using focus group discussions with final year physiotherapy students exposed to a year of CBPHCT. Data from the focus groups were transcribed and analysed using content analysis.

**Results:**

Four overarching themes were identified: prerequisite community-based primary healthcare competencies, positive factors associated with CBPHCT, negative factors associated with CBPHCT and recommendations.

**Conclusion:**

The CBPHCT experience was seen to present challenges to, and have benefits for, physiotherapy students. The students felt that communication between stakeholders, such as academic staff and hospital personnel, could be developed, while the lack of resources, such as Internet access, posed a barrier to learning. Students felt core competencies, such as professionalism of caring, were influenced by their exposure to the clinical personnel. Furthermore, they saw themselves as health advocates and felt there was mutual benefit from engagement with communities during their clinical placements. Recommendations included a review of physiotherapy curricula to prepare students for CBPHCT.

**Clinical implications:**

Community-based primary healthcare clinical training provides learning opportunities for undergraduate physiotherapy students to develop core competencies, such as health advocacy, necessary to address the unique needs of a disparate South African healthcare system.

## Introduction

Community-based primary healthcare clinical training (CBPHCT) is crucial to address the apparent disparity in the distribution of health resources, especially the shortage of healthcare professionals (World Health Organization 2016). Healthcare professionals show a tendency to migrate to urban healthcare facilities, resulting in a distinct lack of healthcare teams serving the disenfranchised and resource-poor regions in South Africa (Mabuza et al. 2013). The public–private healthcare disparity is critical as the majority of South Africans need access to public healthcare services (Kautzky & Tollman 2008). In a bid to remedy this inequality, the South African government introduced the National Health Insurance (NHI) plan so that the majority of South Africans can have access to good healthcare (NHI White Paper 2015). The NHI is dependent on the primary healthcare (PHC) model to provide equal service delivery (PHC Re-engineering 2014). The NHI is in the early stages of development and implementation but seeks to address the inequalities of the South African healthcare system. Community-based primary healthcare clinical training, which is founded on the tenets of PHC, could offer a possible solution to some of the challenges facing the South African healthcare system. The CBPHCT approach advocates for the decentralisation of clinical training for health science students from well-resourced urban facilities to rural and peri-urban, underserved healthcare facilities (Govender et al. 2018).

Possible outcomes of CBPHCT include underserved facilities gaining healthcare from healthcare students as well as the supporting tertiary structures. It is also beneficial for the health science student who has the opportunity to develop core competencies and skills through clinical exposure in a primary healthcare setting (Taukobong 2004).

In 2017, the Kwazulu-Natal Department of Health and the provincial tertiary health science training facilities embarked on a drive to introduce CBPHCT into the health sciences curricula (Govender et al. 2018). The physiotherapy discipline answered the call in 2017 and embarked on the implementation of this novel approach into clinical training. The CBPHCT platform included six extra-clinical training facilities in rural and peri-urban communities, in addition to the existing four urban clinical training hospitals in the eThekwini district. The facilities provided final year physiotherapy students with clinical exposure to neurology, cardiopulmonary, neuro-musculoskeletal conditions and community-based rehabilitation. Each physiotherapy student spent 5 weeks at four of the clinics, two of which were the newly introduced rural/peri-urban facilities, and two of the urban facilities that were used prior to the new CBPHCT approach. Because of the novelty of the new clinical training platform, and the lack of a model to guide clinical training, a group of researchers embarked on a project to develop an integrated model for CBPHCT across the disciplines in the health sciences (Govender et al. 2018). The aim of this study was specifically to explore the perceptions of final year undergraduate physiotherapy students about CBPHCT so that their insights could be incorporated into the integrated model for CBPHCT in health sciences at the same university.

## Materials and methods

Using an explorative qualitative approach (Baxter & Jack 2008), the perceptions of physiotherapy students participating in the CBPHCT offered at a tertiary institution were explored. Focus group discussions were the primary method of enquiry. This approach allowed for a comprehensive understanding of the experiences of students accessing the clinical training (Wildemuth 2016). The study was located at the University of KwaZulu-Natal. A purposive sample of final year physiotherapy students, who were exposed to the training for a year, was used to gain insight into their experiences of the new clinical training approach (Onwuegbuzie et al. 2009). The sample comprised both male and female students of varying ages and ethnicities representative of all the rural and urban clinical sites to achieve maximum variation. The power influences of interviewer–interviewee relations were managed by electing a part-time staff member (part of the research team) who was not involved in final year clinical training to conduct the interviews together with a staff member from the student academic support services.

Four focus group discussions were conducted by the first author and observed by a senior academic at the university, who is experienced in qualitative methodology (Gill et al. 2008). The discussions were conducted in a meeting room in the physiotherapy department and lasted between 45 and 60 min. Informed consent and biographical information were obtained from the students and they were made aware that they could withdraw from the study at any point. No incentives were offered to participate in the study (Gill et al. 2008). A focus group guide developed by the authors was used as a basis for understanding students’ experiences during their clinical placements. The questions included perceptions of being the pioneers of the CBPHCT at the institution, experiences of the four mandatory clinical training sites attended by students in the year and also explored relationships on the CBPHCT platform including peer support. Facilitators and barriers as they emerged were also probed. Students were also asked about their perceptions of the teaching and learning approaches to CBPCHT, as well as their recommendations for an approach to teaching and learning on the CBPHCT platform.

Discussions were conducted in English, which was the preferred language of the students, and were recorded using a digital voice recorder. Data were collected until no new information emerged from the discussion. The recordings were transcribed verbatim immediately after the focus group discussions. The observer, an academic development officer who is part of the student support service, documented non-verbal cues to contribute to the credibility of the verbally expressed data. Then, conventional thematic data analysis was conducted by using an inductive approach (Vaismoradi, Turunen & Bondas 2014). Consensus was reached following reading, analysis and in-depth discussions among the co-authors of the study. This process ensured the dependability of the data. The themes and subthemes that emerged were sent back to students for verification of the results as a means of member checking to enhance the confirmability of the data (Morse et al. 2002). Detailed, rich descriptions further contributed to the transferability and credibility of the data (Creswell & Miller 2000).

### Ethical consideration

Ethical clearance was obtained from the Humanities and Social Sciences Research Ethics Committee of the University of KwaZulu-Natal (ethical clearance number: HSS/0576/018M).

## Findings

There were 39 female and 10 male physiotherapy students who participated in the study. Of the students, 47 were aged between 20 and 25 years and two were aged between 26 and 30 years. Of the students, 28 were black South Africans, 17 were of Indian descent and 4 were white South Africans. Four overarching themes were identified from the focus group discussions: prerequisite competencies for CBPHCT, positive factors associated with CBPHCT, negative factors associated with CBPHCT and recommendations, which are discussed next.

Theme 1, *prerequisite competencies for CBPHCT*, was made up of five categories: community respect, cultural sensitivity, language competency, health advocacy and professionalism. The students learnt the importance of community respect and to be culturally sensitive, as well as the need to improve their language competency during their community-based primary healthcare clinical training. Physiotherapy students embraced their role as health advocates and felt that the training influenced their professional approach to care.

Theme 2, *positive factors associated with CBPHCT*, with the subtheme *benefits of the clinical training platform*, included three categories: personal development, clinical support and independent learning. The support offered by clinicians in this study, through tutorials and mock examinations, enhanced the clinical training. The rural placements challenged students to develop their independent learning skills.

Theme 3, *negative factors associated with CBPHCT*, included four subthemes: logistical factors with six categories (poor infrastructure, online learning access, lack of consumables and equipment, accommodation challenges, administrative challenges and transport problems), curriculum gaps with three categories (theoretical gaps, assessment and theory into practice), collaborative influence on learning with four categories (academic and clinician dynamics, student and clinician partnership, peer relationships and poor multidisciplinary team work) and obstacles to clinical training platform with three categories (lack of knowledge, lack of supervision and inadequate specialised exposure).

Theme 4, *recommendations*, included three categories: personal development, clinical support and independent learning. Physiotherapy students in this study recommended that the infrastructure needs to be improved. They felt that access to computers, printers and Wi-Fi would facilitate their learning. Furthermore, physiotherapy students believed that improved accommodation at the rural and peri-urban placements would enhance their CBPHCT experience. Table 1 shows relevant quotes, categories and themes. Pseudonyms are used when quoting the students.

**TABLE 1:** Themes, subthemes, categories and illustrative quotes.

Categories	Quotes
**Theme 1: Prerequisite competencies for community-based primary healthcare**	
Community respect	‘People in the rural areas are very appreciative. They get so happy when you walk into the ward’ (Charlotte, 22 years old, white)
	‘The community wants you there to help them, they respected us’. (Priya, 21 years old, Indian)
	‘The community was so grateful for having help and assistance’. (Xolani, 23 years old, black)
Cultural sensitivity	‘I learnt what the meaning of Ubuntu really is. I thought being a Zulu-speaking student I knew, but I didn’t’. (Musi, 26 years old, black)
	‘It was very interesting to learn how things work and what people believe, like culture stuff, I mean’. (Nivaar, 24 years old, Indian)
Language competency	‘You are exposed to a lot more languages in DCT (Decentralised Clinical Training) and learn so much from the community’. (Zanele, 23 years old, black)
	‘It helped my language grow. The patients would try to teach us new words’. (Santhuri, 25 years old, Indian)
	‘It made me want to learn more Zulu so I could communicate with my patients’. (Charlotte, 22 years old, white)
Health advocacy	‘They really appreciated the health education we gave them and they wanted to change how they eat and stuff (referring to community health education projects)’. (Thandeka, 25 years old, black)
	‘Patients learnt a lot about the different types of TB (tuberculosis) strains and we even taught them precautions’. (Annette, 22 years old, Indian)
Professionalism	‘The nurses at DCT (decentralised training sites) were very professional and pleasant to work with, so I learnt a lot from them’. (Musi, 26 years old, black)
	‘Everybody worked together to treat the patients and it helped me with approaching my patients’. (Zanele, 23 years old, black)
**Theme 2: Positive factors associated with CBPHCT** **Subtheme: Benefits of clinical training platform**	
Personal development	‘DCT really built you as a person. You broaden your perspective’. (Letisha, 22 years old, Indian)
	‘It makes you begin to think like an adult. Taught us how to budget’. (Zama, 24 years old, black)
	‘You could see you can make it on your own’. (Mabusa, 25 years old, black)
	‘It taught me to work with different people of different personalities’. (Tracy, 24 years old, Indian)
Clinician support	‘The staff at DCT were very supportive. They gave us advice on our projects’. (Pam, 23 years old, Indian)
	‘The physio at one DCT hospital would do mock exams and ask us to submit assessments’. (Zama, 24 years old, black)
	‘Advantage of going away is the good wholesome supervision in community’. (Thembi, 26 years old, black)
	‘We got the best supervision at a DCT site’. (Thobani, 23 years old, black)
	‘There was very good supervision at rural sites compared to urban’. (Adele, 21 years old, Indian)
Independent learning	‘You take responsibility and put learning into your own hands’. (Tracy, 24 years old, Indian)
	‘If you don’t know something, then you have to research and it helps you to be confident because you realise you can do it’. (Priya, 21 years old, Indian)
	‘Your skills improve because you are treating a lot of different conditions’. (Letisha, 22 years old, Indian)
	‘Rural areas helped me to develop my skills and how to create my own equipment as a physiotherapist’. (Tanya, 20 years old, Indian)
	‘You have to be creative because you have nothing to work with so make the best with what you have and make it work’. (Charlotte, 22 years old, white)
**Theme 3: Negative factors associated with CBPHCT** **Subtheme: Logistical factors**	
Poor infrastructure	‘Some of the physio departments are really small. You can only treat one patient at a time’. (Thiru, 24 years old, Indian)
	‘We were very busy in some hospitals because there were few staff’. (Nosipho, 27 years old, black)
Online learning access	‘We would run out of data using the router’. (Nivaar, 24 years old, Indian)
	‘We only had 5 gigabytes and that was used in one week’. (Xolani, 23 years old, black)
	‘We did not have access to Wi-Fi very often’. (Diane, 21 years old, white)
Lack of consumables and equipment	‘DCT was financially challenging with little resources available for patients’. (Sandy, 21 years old, Indian)
	‘DCT needs a lot of money to support the clinics’. (Zakhele, 28 years old, black)
Accommodation challenges	‘Accommodation was horrible and was very dirty’. (Tanya, 20 years old, Indian)
	‘Our beds had bed bugs and we couldn’t sleep’. (Charlotte, 22 years old, white)
	‘Accommodation was not appropriate for learning. People would play loud music’. (Revashni, 23 years old, Indian)
Administrative challenges	‘Better planning could have worked to our favour like accommodation and travel’. (Thandinkosi, 26 years old, black)
	‘The whole process leading to us leaving was very disorganised from the university side’. (Brandon, 25 years old, Indian)
Transport problems	‘The drivers refused to take us to the clinics or shopping but they were being paid for those services’. (Mumtaz, 20 years old, Indian)
	‘Transport issues were a big problem’. (Charlotte, 22 years old, white)
**Subtheme: Curriculum gaps**	
Theoretical gaps	‘You can have a lecture once a week, even a recording because Wi-Fi is so bad’. (Xolani, 23 years old, black)
	‘The university staff should do more tutorials. They should Skype’. (Letisha, 22 years old, Indian)
Assessment	‘We were not given time to do assessments because we had so many patients to see’. (Nosipho, 27 years old, black)
	‘They gave us 10 patients a day. You do not get time to read on everything you have seen. You don’t have time to assess’. (Priya, 21 years old, Indian)
Theory into practice	‘Not everything can be learnt from a book, things we learn in clinicals cannot be taught in a lecture room’. (Tracy, 24 years old, Indian)
	‘Clinicals should start in 2nd year; clinicals teaches us how to handle yourselves and how to handle the patient. That is something you cannot learn in a classroom’. (Sandile, 22 years old, black)
**Subtheme: Collaborative influence on learning**	
Academic and clinician dynamics	‘Communication with the university staff and hospital staff is very slow’. (Diane, 21 years old, white)
	‘ Urban clinicians would blame the lecturers saying that they threw us into the deep end’. (Tanya, 20 years old, Indian)
	‘University should discuss with the health department and physiotherapy managers about all of the plans’. (Brandon, 25 years old, Indian)
Student and clinician partnership	‘Clinicians took advantage of us (referring to urban sites later in the narrative)’. (Zakhele, 28 years old, black)
	‘Clinicians should not leave it to the students to do their work’. (Revashni, 23 years old, Indian)
	‘Clinicians did not want to supervise because they said they don’t get paid to supervise’. (Pam, 23 years old, Indian)
	‘Clinicians in Durban did not like students. They hate students. We are their slaves’. (Roxanne, 22 years old, Indian)
	‘Urban clinicians expect us to know everything’. ‘They did not even greet us’. (Zanele, 23 years old, black).
Peer relationships	‘You stayed with people who you never thought you could be friends with. DCT brought that’. (Pam, 23 years old, Indian)
	‘It was peer support all the way that helped me get through DCT’. (Adele, 21 years old, Indian)
	‘We were all in the same boat so there was a lot of peer support’. (Thobani, 23 years old, black)
	‘We were supportive of each other and lived as a family’. (Diane, 21 years old, white)
Poor multidisciplinary teamwork (MDT)	‘They did their own thing and we did our own thing (referring to urban clinicians)’. (Cris, 22 years old, Indian)
	‘I felt the MDT was quite non-existent in urban sites’. (Sandile, 22 years old, black)
	‘The doctors don’t even look at you when you’re in the ward’. (Diane, 21 years old, white)
**Subtheme: Obstacles to clinical training platform**	
Lack of perceived knowledge	‘You did not feel like we were going out as a professional physiotherapist knowing everything’. (Nosipho, 27 years old, black)
	‘We had to be independent too soon. We didn’t have the practical experience to handle every clinical situation’. (Lyn, 22 years old, Indian)
Lack of supervision	‘One of the challenges in DCT was that we did not have enough supervision from university staff’. (Charlotte, 22 years old, white)
	‘Urban clinicians didn’t want to supervise because they said they don’t get paid for that’. (Pam, 23 years old, Indian)
	‘They just left us alone with no supervision’. (Thulani, 25 years old, black)
Inadequate specialised exposure	‘We need to have a paediatric block’. (Diane, 21 years old, white)
	‘We did not get enough exposure to neurology patients because we were too busy treating patients in outpatients’. (Pam, 23 years old, Indian)
	‘There was not many ICU patients in some hospitals’. (Nosipho, 27 years old, black)
**Theme 4: Recommendations**	
Improved communication	‘Communication with university staff and hospital staff needs to improve’. (Andiswa, 30 years old, black)
	‘Department should be in constant communication with the transport department’. (Santhuri, 25 years old, Indian)
	‘University should explain to clinicians that students can see other patients but must mostly see what the block is for’. (Snobile, 21 years old, black)
Increased clinical supervision	‘They should have mock exams. It is amazing and helps to prepare you’. (Andiswa, 30 years old, black)
	‘We need supervision at least once a week’. (Adele, 21 years old, Indian)
	‘We need more supervision in urban hospitals because clinicians are not willing to help’. (Thulani, 25 years old, black)
	‘If the clinicians are willing to teach you, then there would be no need for the lecturers to come so often’. (Thandeka, 25 years old, black)
Improved infrastructure	‘There should be unlimited Wi-Fi’. (Nivaar, 24 years old, Indian)
	‘There should be park homes with computers and printers at all DCT sites’. (Snobile, 21 years old, black)

CBPHCT, Community-based primary healthcare training.

## Discussion

The perceptions of physiotherapy students about the CBPHCT approach offered at the university who participated in this study are discussed under the four core themes and framed by contemporary literature.

The *prerequisite competencies for community-based primary healthcare* assumes that community-based primary healthcare competencies are embedded in community respect, cultural sensitivity, language competency, health advocacy and professionalism. South African universities promote graduate competencies to prepare their undergraduate students for professional practice upon completion of their degree.

The framework that guides the training of health science students in this study setting espouses seven key roles: practitioner, communicator, collaborator, leader, scholar, health advocate and professional (Chetty et al. 2018; Govender et al. 2018). The core competencies align with the perceived competencies that physiotherapy students in this study identified after their exposure to the CBPHCT model. Future healthcare professionals, such as physiotherapists, need to develop social responsibility and professional attributes that make them community leaders and innovators. Mlambo et al. (2018) suggested that decentralised clinical training, such as the CBPHCT, offers an opportunity for health science students to learn these competencies and become socially responsive during their undergraduate clinical training.

The community respect experienced by the students in this study facilitated their learning experience. This finding is supported by Mabuza et al. (2013), where the students were deemed vital members of the healthcare team by the community, and where it was acknowledged that communities were key to educating prospective healthcare professionals (Diab & Flack 2013). In a study regarding community-based education, health science students in South Africa agreed that exposure to diverse communities facilitated the development of students’ cultural awareness and sensitivity. Focusing on language competency, physiotherapy students felt motivated to improve their local language skills. In their 2013 study exploring clinical training with physiotherapy undergraduate students at a South African tertiary institution, Mostert-Wentzel and colleagues believed that understanding local languages facilitates the relationship between health science students and the community (Mostert-Wentzel, Frantz & Van Rooijen 2013).

The students indeed have perceived CBPHCT positively. This is evident from the positive factors associated with CBPHCT. This core phenomenon subsumes the benefits to the clinical training platform and asserts that personal development, clinician support and independent learning are facilitators of the CBPHCT platform.

Physiotherapy students in our study stated that they had grown on both a personal and a professional level. Two studies at South African universities, focusing on undergraduate physiotherapy students, concurred that students develop personally and professionally during their clinical placements (Ernstzen, Statham & Hanekom 2014; Talib et al. 2017). Respondents in this study stated that the clinicians at the rural and peri-urban facilities engaged more, and offered more support as supervisors, than the clinicians at the urban sites. Naidoo and Van Wyk (2016) expressed the opinion that health science students developed their clinical skills through observing their mentors. They found themselves having to be creative and resourceful in poorly resourced environments and took responsibility for their learning. Ernstzen et al. (2014) and Taukobong (2004) found that the challenges at rural clinics facilitated physiotherapy students to think laterally and develop their clinical reasoning skills.

In spite of the benefits perceived by physiotherapy students, there were still some negative factors associated with CBPHCT, which included logistical factors, collaborative influence on learning, obstacles to clinical training platforms and curriculum gaps. Logistical factors included the categories of poor infrastructure, online learning access, lack of consumables and equipment, accommodation challenges, administrative challenges and transport problems. The physiotherapy students stated that the poor infrastructure and lack of resources influenced their learning. In a similar South African study, Ernstzen et al. (2014) opined that limited infrastructure can have both negative and positive effects on clinical learning of undergraduate physiotherapy students. A recurring challenge for our students was inadequate Internet access during their rural and peri-urban placements. In Talib et al.’s (2017) study, with medical students in sub-Saharan Africa, learning was hindered because of limited resources in the learning environment, such as Internet access. Although limited resources will present challenges in poor- and middle-income countries like South Africa, these obstacles encourage students to be creative and solve problems (Ernstzen et al. 2014).

Collaborative influences on learning encompassed academic and clinician dynamics, student and clinician partnerships, and peer relationships. Physiotherapy students believed that the partnership between the academic staff at the university and the staff at the CBPHCT sites was crucial for the success of learning in this model. In a similar study conducted prior to the adoption of CBPHCT, students reiterated that poor communication between the university and clinical training sites can be an obstacle to optimal learning (Chetty et al. 2018). Diab and Flack (2013) also attest that universities should not only train the clinicians supervising health science students but also provide them with ongoing support. The physiotherapy students voiced that clinicians employed at the urban facilities were unwilling to offer supervision or assist them with their teaching and learning. The relationship between the student and clinician is critical and the clinicians need to be approachable and dynamic to enable a favourable learning environment (Ernstzen, Bitzer & Grimmer-Somers 2010). The physiotherapy students expressed dissatisfaction about the multidisciplinary teamwork, especially at the urban clinics. They stated that the doctors did not acknowledge their role. In Mostert-Wentzel et al.’s (2013) study, physiotherapy students stated that doctors lacked awareness of physiotherapy and/or insight into allied health professions. Multidisciplinary teamwork (MDT) is very important if seamless healthcare is to be offered and the issue of collaboration should be explored further, especially regarding CBPHCT (Chetty et al. 2018; Ernstzen et al. 2014).

A lack of perceived knowledge, lack of supervision and inadequate specialised exposure posed barriers to the clinical training platform. Some physiotherapy students felt unprepared to practise independently and stated that they did not have adequate knowledge. They said that they lacked supervision and exposure to specialised areas of practice, such as neurology and paediatrics. In a similar South African study, an explorative review of physiotherapists doing their community service found that the respondents agreed with these sentiments, but in retrospect, once they had experienced independent practice, found the undergraduate training adequate (Chetty et al. 2018).

What is adequate supervision for physiotherapy undergraduate students? This is a relevant question, yet there is little in the literature which provides an answer. A recent systematic review by Lekkas et al. (2007) concluded that no model of clinical education is superior to any other. Taukobong (2004) reported that students require suitable supervision to enable a positive learning environment. The lack of specialisation is echoed in previous studies by Ramklass (2013) and Chetty et al. (2018), and these shortcomings need to be addressed in order to facilitate the preparedness of physiotherapy students prior to clinical training.

The curriculum gaps included assessment and theory into practice gaps. Physiotherapy students felt that they needed more input from the academic staff at the university in the form of tutorials and Skype sessions.

Our respondents stated that they were unable to complete assessments because of the demands of patient care placed on them. In Ernstzen et al.’s (2014) study, physiotherapy students agreed that the demands of heavy workloads encroached on assessment time and therefore affected clinical learning. Our physiotherapy students said that the current programme of clinical exposure should begin earlier in the curriculum, and not in level 3, in order to better integrate theory into practice. Talberg and Scott (2014), assessing a physiotherapy undergraduate programme in South Africa, noted that undergraduate students struggle to integrate theory into practice. Introducing clinical education earlier in the programme could alleviate this problem.

Finally, the recommendations included improved communication, increased clinical supervision and improved infrastructure. The physiotherapy students stated that communication needs to improve between academic and clinical staff in order to improve learning. Mabuza et al. (2013), in a study of health science students, highlighted the impact of poor communication in hindering clinical training. Students suggest that, prior to clinical placements, university staff should ascertain from clinical supervisors whether they are willing to supervise students. Physiotherapy students in Ernstzen et al.’s (2010) study on the perspectives of physiotherapy students and clinical teachers regarding best clinical teaching and learning reported that they learnt through observation and that their personal clinical reasoning developed as the clinical supervisors shared their clinical reasoning with them. Ernstzen et al.’s study validated the powerful role of clinical supervisors in the teaching and learning of physiotherapy students.

## Limitations

While the results of this study may be transferable to other contexts, they are not generalisable. The perceptions of physiotherapy students about CBPHCT are specific to their own experiences at one university and thus cannot be generalised to the broader population of South African physiotherapy students. Furthermore, although reflexivity was taken into account, the authors note the potential for internal bias in the interpretation of the findings because of their positions at the institution.

## Recommendations

Future studies exploring the collaboration between clinical educators and undergraduate students in this context need to be conducted in order to provide insights into and recommendations for improving partnerships.

Furthermore, a review of the undergraduate curriculum is important to include community-based clinical training.

## Conclusion

Improved approaches to clinical training for healthcare professionals, such as physiotherapists, in resource-poor countries like South Africa are needed to address the healthcare needs of the country. The CBPHCT platform introduced at a tertiary level allows students to be exposed to rural and underserviced communities during their clinical training. This study explored the experiences of final year physiotherapy students who participated in the recently introduced CBPHCT at the University of KwaZulu-Natal. Physiotherapy students experienced challenges during their CBPHCT placements, such as curriculum gaps, lack of adequate communication between stakeholders and limited resources. However, the benefits they derived included developing more competence in areas such as health advocacy and improved professionalism. The physiotherapy students experienced personal and professional growth and felt that they were contributing to the communities they engaged with during their clinical placements. The CBPHCT approach was seen as a feasible method to address various needs of undergraduate health science training while providing care at a primary healthcare level.
